# The PPAR-gamma agonist pioglitazone protects cortical neurons from inflammatory mediators via improvement in peroxisomal function

**DOI:** 10.1186/1742-2094-9-63

**Published:** 2012-04-05

**Authors:** Elizabeth Gray, Mark Ginty, Kevin Kemp, Neil Scolding, Alastair Wilkins

**Affiliations:** 1 Multiple Sclerosis and Stem Cell Group, Burden Centre, Institute of Clinical Neurosciences, Frenchay Hospital, University of Bristol, Bristol BS16 1JB, UK

**Keywords:** Peroxisome, Nitric oxide, Pioglitazone, Peroxisome proliferator activated receptor

## Abstract

**Background:**

Inflammation is known to play a pivotal role in mediating neuronal damage and axonal injury in a variety of neurodegenerative disorders. Among the range of inflammatory mediators, nitric oxide and hydrogen peroxide are potent neurotoxic agents. Recent evidence has suggested that oligodendrocyte peroxisomes may play an important role in protecting neurons from inflammatory damage.

**Methods:**

To assess the influence of peroxisomal activation on nitric oxide mediated neurotoxicity, we investigated the effects of the peroxisomal proliferator activated receptor (PPAR) gamma agonist, pioglitazone in primary cortical neurons that were either exposed to a nitric oxide donor or co-cultured with activated microglia.

**Results:**

Pioglitazone protected neurons and axons against both nitric-oxide donor-induced and microglia-derived nitric oxide-induced toxicity. Moreover, cortical neurons treated with this compound showed a significant increase in the protein and gene expression of PPAR-gamma, which was associated with a concomitant increase in the enzymatic activity of catalase. In addition, the protection of neurons and axons against hydrogen peroxide-induced toxicity afforded by pioglitazone appeared to be dependent on catalase.

**Conclusions:**

Collectively, these observations provide evidence that modulation of PPAR-gamma activity and peroxisomal function by pioglitazone attenuates both NO and hydrogen peroxide-mediated neuronal and axonal damage suggesting a new therapeutic approach to protect against neurodegenerative changes associated with neuroinflammation.

## Background

Axon injury and neuronal loss are major pathological substrates for permanent neurological disability in many neurological diseases [1]. In several central nervous system disorders activated inflammatory cells produce large quantities of reactive oxygen species (ROS) and nitrogen species (RNS) such as superoxide, hydrogen peroxide and nitric oxide (NO) that can oxidize and damage proteins, nucleic acids and lipids leading to mitochondrial damage [2] with associated neuronal injury and axonal degeneration [3]. In particular, microglia-derived NO has been shown to be neurotoxic *in vitro *[4,5] and our recent work has demonstrated that microglia-derived NO significantly reduces the number of phosphorylated neurofilaments within axons, leading to structural instability and ultimately axonal degeneration [6,7].

The detoxification of ROS through the action of antioxidant enzymes such as superoxide dismutase and catalase, is a major intrinsic defense mechanism against inflammatory tissue damage. Catalase is predominantly located in peroxisomes where it catalyzes the conversion of hydrogen peroxide into water and molecular oxygen [3]. As well as performing an important role in the detoxification of ROS, peroxisomes are also responsible for the synthesis of plasmalogens and β-oxidation of very long chain fatty acids (VLCFAs) [8]. Interestingly, abnormalities in peroxisomal function have been linked to a variety of neurological disorders including the inflammatory demyelinating disorder, X-linked adrenoleukodystrophy (X-ALD). Recent evidence has demonstrated that peroxisomes appear to be indispensible within oligodendrocytes for the maintenance of myelin and for the integrity of axons [9,10] as oligodendrocyte restricted elimination of peroxisomes is associated with axonal damage, neuroinflammation and subcortical demyelination [10]. Furthemore, an association between neuroinflammation and impaired peroxisomal function has also been demonstrated in a model of experimental autoimmune encephalomyelitis [11].

Peroxisome proliferator-activated receptor-γ (PPAR-γ) is a ligand-activated nuclear transcription factor [12] that is predominantly expressed in adipose tissue, the immune system [13] and also in primary rat microglial [14] and neuronal cultures [15]. It is a target of the class of drugs known as thiazolidinediones (TZDs), used to treat type II diabetes and is known to regulate lipid and carbohydrate metabolism [16-18] and act as a negative regulator of macrophage and microglial activation [14,19,20]. More recently, PPAR-γ agonists have received considerable attention as potential therapeutic agents for a wide range of neurological diseases, including neurodegenerative diseases, traumatic injuries, stroke and demyelinating diseases [21,22]. Indeed, several studies have indicated that PPAR-γ ciaos can prevent or attenuate neurodegeneration [23-25] and have beneficial effects in the amelioration of experimental autoimmune encephalomyelitis (EAE) [26-31] which could be explained in part because of anti-inflammatory action exerted through PPAR-γ activation in glial cells [14,32-34]. PPAR-γ can activate genes with a peroxisome proliferator response element (PPRE) in their promoter regions [35]. Indeed, the catalase promoter is known to contain functional PPAR-γ responsive elements, so it is possible that the activity of catalase could be regulated by PPAR-γ agonists [36].

In our study, we evaluated the neuroprotective properties of the PPAR-γ agonist pioglitazone on cortical neurons exposed to inflammatory mediators, and assessed whether pioglitazone influences axonal morphology and peroxisomal function. Our experiments show that pioglitazone is capable of protecting cortical neurons from the NO donor DETANONOate, hydrogen peroxide and from microglia-derived injury. Pretreatment with pioglitazone was also observed to increase the total levels of phosphorylated neurofilament within axons. Furthermore, we provide evidence that pioglitazone can induce the expression of PPAR-γ and the activity of catalase, a key peroxisomal enzyme. These findings suggest that activation of PPAR-γ protects cortical neurons against NO and hydrogen peroxide-dependent toxicity by a mechanism that involves the enhancement of endogenous peroxisomal function.

## Materials and methods

### Materials

The PPAR-γ agonist, pioglitazone was purchased from Cayman Chemical (Ann Arbor, Michigan, USA) and the PPAR-γ antagonist, GW9662, the catalase inhibitor, 3-aminotriazole (3-AT) and catalase extracted from human erythrocytes were obtained from Sigma-Aldrich (Gillingham, UK). Pioglitazone and GW9662 were dissolved in dimethyl sulfoxide (DMSO) to reach the stock concentration of 100 mM and serial dilutions were prepared using DMSO and stored in aliquots at −20°C. Final working concentrations of 10 μM, 1 μM and 0.1 μM were used in minimal media (DMSO, final concentration 0.1%, V/V). A vehicle control with a final concentration of 0.1% DMSO (v/v) was used alongside each experiment. 3-AT was dissolved in sterile distilled water to reach the concentration of 1 M. Final working concentration of 10 mM 3-AT was used in minimal media.

### Neuronal cell culture

Neuronal cultures were prepared from the cortices of E18 rat embryos as previously described [37]. Following enzymatic and mechanical dissociation, cells were counted and plated onto poly-L-lysine coated 13 mm coverslips at 250,000 cells/coverslip or onto poly-_L_-lysine coated 96 well plates at 100,000 cells/well and cultured in (D)MEM supplemented with 2% B27 (Gibco, Paisley, UK) and 1% penicillin/streptomycin. After five days *in vitro *(DIV), >95% of cells were positive for the neuronal marker β-tubulin. At this point, cultures were exposed to experimental conditions. The base medium for all experiments was 'minimal', which consisted of (D)MEM supplemented with insulin-free Sato (containing 100 μg/ml BSA), 100 μg/ml transferrin, 0.06 μg/ml progesterone, 16 μg/ml putrescine, 0.04 μg/ml selenite, 0.04 μg/ml thyroxine, 0.04 μg/ml triiodothryonine).

### Microglial cultures

Mixed glial cultures were prepared following the protocol of McCarthy and deVellis (1980), as described previously [37]. Briefly, forebrains of new-born (P2) rat pups were removed and the meninges stripped before mechanical and enzymatic dissociation. The resulting cell suspension was plated onto poly-_L_-lysine coated 75 cm^2 ^tissue culture flasks. Culture medium ((D)MEM plus 10% fetal calf serum) was changed at 24 hours and twice weekly thereafter until the cells reached confluence after 10 to 12 days. At this stage, the loosely adherent microglia on top of a confluent glial cell layer were harvested by shaking at 700 rpm for 60 minutes. After centrifugation (2,000 rpm for five minutes), cells were re-suspended in serum-free (D)MEM supplemented with 2% B27 (Gibco, Paisley, UK) and cultured in transwell inserts (Millipore, Watford, UK) at 100,000 cells per well for 24 hours in (D)MEM supplemented with 2% B27 (Gibco) and 1% penicillin/streptomycin (B27 medium), prior to being exposed to experimental conditions. The final supernatant from these shaken cultures contained approximately 90% microglia as determined by immunocytochemistry using antibodies to OX-42 (Abcam, Cambridge, UK)

### Co-cultures of microglia and cortical neurones

Microglia within transwells were cultured in 2% B27 media for 24 hours. At this point, the transwells inserts were added to cortical neuronal cultures (in 24-well plates at 250,000 cells per well) already maintained for five days *in vitro *and cultures were incubated in serum-free defined medium for two days with or without Lipopolysaccharide (LPS)(1 μg/ml)/interferon (IFN) γ (100 U/ml) and pioglitazone (1 μM or 10 μM). DMSO was used as a vehicle control since it was used to dissolve pioglitazone.

### NO_2_^- ^determination

NO_2_^- ^levels, measured with the Griess reagent, were taken as an estimate of NO production. Griess reagent (Sigma) was added to an equal volume of cell culture supernatant (100 μl) and incubated for 20 minutesat room temperature. The optical density was measured at 570 nm and a standard curve established using NO_2_^- ^at a range of 1 to 100 μM.

### Assays for cell survival

At five days *in vitro *culture, cortical neuronal cells were exposed to experimental conditions. Media was removed from all wells and cells were washed twice in (D)MEM. (D)MEM/2% B27 medium (1 ml) or minimal media (1 ml) was added to appropriate wells. Pioglitazone, at the concentrations described above was added one hour before treatment with an NO donor. The nitric oxide donor DETANONOate (0.1 mM) was applied to cultures for 24 hours.

Evaluation of cortical neuronal cell survival was carried out using 3-(4,5-dimethyl-thiazol-2-yl)-2,5-diphenyltetrazolium bromide (MTT) assays, immunocytochemistry and morphological examination of neuronal cultures using the following methods.

### MTT assay

The viability of cell cultures was estimated using MTT (Sigma). This tetrazolium dye is oxidized by mitochondrial dehyrdrogenases and is a sensitive marker for mitochondrial function. Cells were incubated with MTT (1 mg/ml in PBS) for one hour at 37°C. Following this incubation, the MTT solution was removed from the plate and left to dry. DMSO (200 μl) was then added to each well to dissolve the formazan crystals before the optical density was measured at 540 nm.

### Immunocytochemistry

Immunocytochemistry was used to identify cell phenotypes and allowed examination of cellular morphology, and PPARγ expression. Neuronal cultures were stained after fixation with 4% paraformaldehyde. Primary antibodies against intracellular markers were used after treatment of fixed cultures with 100% methanol for ten minutes at −20°C. These were β-III tubulin (1:600) (Sigma-Aldrich), SMI312 (phosphorylated neurofilament; 1:1000) (Covance, Cambridge, UK), and PPARγ (1:200) (Santa Cruz, Heidelberg, Germany). Species specific (1:500) Alexa Fluor® 488 and 555 conjugated secondary antibodies (Invitrogen, Paisley, UK) were used to visualize primary antibody staining. 4',6-Diamidino-2-phenylindole (DAPI) Vectashield™ {Vector Laboratories, Peterborough, UK was used for nuclear identification.

### Morphological analysis using immunocytochemistry

Neuronal cells were identified by β-III tubulin expression. In addition, nuclear staining (DAPI) of cells enabled a morphological assessment of apoptosis. Neuronal survival was assessed using counts of live β-III tubulin positive stained cells as the 'gold standard'. These cultures exhibited a lack in apoptotic cell death as evidenced by fewer condensed, fragmented nuclei. In all cases, a control culture of cells grown throughout the experimental period in (D)MEM/insulin-free Sato medium referred to as 'MIN' was also analyzed and values for experimental conditions were divided by this value to standardize results between experiments.

### Analysis of axons in culture

The antibody SMI312 (Covance) labels phosphorylated neurofilaments and can be used to distinguish axons from dendrites [38]. Cultures were stained for SMI312 after fixation and were viewed under a fluorescent microscope with digital images (x40) taken of five random fields within each culture (at least three culture conditions per experiment). The images were analyzed using Image J (NIH) and axon length was measured in arbitrary units. Phosphorylated axons identified by SMI312 immunoreactivity were traced using the freehand line tool. From the 'set measurements' function in the tool bar, the length box was checked. Lengths of processes staining positive for SMI312 per field were obtained, together with the number of live cells per field.

### Western blotting

Neurons were cultured at high density (2 × 10^6 ^cells/well) in a six well plate for five days before exposure to test conditions. After 24 hours, cells were lysed using Beadlyte cell signalling universal lysis buffer (Millipore, Watford, UK). The Quant-iT™ Protein kit (Invitrogen, UK) was then used to quantify the concentration of total protein within each cell lysate sample according to manufacturer's instructions to ensure equal loading of cell lysates. Lysates were heated to 95°C for five minutes with Laemmli 2 × sample buffer (Invitrogen) and run on Tris-HCl 10 to 20% ready gels (Bio-Rad, Hemel Hemsptead, UK,). After transfer to nitrocellulose membrane (Bio-Rad) and blocking in 5% w/v BSA, membranes were incubated overnight in primary antibody at 4°C (in Tris-buffered saline/5% BSA). Antibodies used were PPARγ (1:5000) (Abcam) and GAPDH (1:15000) (Abcam). Immunoreactivity was detected using secondary anti-rabbit and anti-mouse horseradish peroxidase conjugated antibodies (Abcam) (in Tris-buffered saline/5% BSA) and specific protein expression patterns were visualized by chemiluminescence using a Geneflow Limited Western Blotting Detection System (Geneflow Limited, Staffordshire, UK). The optical density of the bands (integrated density, arbitrary units) was measured by Image J and levels of expression were calculated relative to MIN control.

### Catalase assay

Catalase activity was measured using the amplex® red catalase assay kit (Invitrogen). In the assay, catalase activity is indirectly proportional to resorufin activity. Neurons were cultured at high density (25 × 10^6 ^cells per flask) in T175 flasks for five days before exposure to test conditions. After 24 hours, cells were collected in ice-cold PBS by scraping using a rubber policeman and were pelleted by centrifugation at 2,000 g for 10 minutes at 4°C. Following removal of the supernatant, the pellet was homogenized in 50 mM phosphate buffer, pH 7 containing 1 mM ethylenediaminetetraacetic acid (EDTA) and subsequently centrifuged at 10,000 g for 15 minutes at 4°C. Supernatants were stored in aliquots at −80°C prior to being assayed for catalase activity following the manufacturer's instructions. The assay can detect catalase in a purified system at levels as low as 50 mU/mL. All samples were analyzed in triplicate.

### Real time PCR

Neurons were cultured at high density (2 × 10^6 ^cells/well) in six well plates for five days before exposure to test conditions. To investigate the effect of nitric oxide and/or pioglitazone on gene expression, cells were treated with either vehicle or pioglitazone (1 μM) in combination with DETANONOate (0.1 mM) for one, two and four hours. At each time point, RNA was extracted and cDNA produced using the TaqMan gene expression cells-Ct-kit (Applied Biosystems Paisley, UK) according to the manufacturer's instructions. A total of 10^5 ^cells were added to the lysis solution plus DNase 1 (five minutes) before the addition of the stop solution (two minutes). The total RNA concentration was measured using Quant-iT™ RNA kit (Invitrogen, UK). To synthesize cDNA, the extracted RNA was placed in a thermal cycler with the RT buffer and RT enzyme mix and incubated at 37°C for one hour and 95°C for five minutes. Real-time RT-PCR was performed using the Step One Plus system (Applied Biosystems), with assay-on-demand gene expression products for PPAR-γ, neuron-specific enolase (Taqman MGB probes, FAM dye-labeled, Applied Biosystems) and 18 S (VIC dye-labeled, Applied Biosystems), gene Expression master mix and 2.5 ng of cDNA in a total volume of 20 μl: 50°C for 2 minutes; 95°C for 10 minutes; and 40 cycles of 95°C for 15 minutes and 60°C for 1 minute. All samples were analyzed in triplicate. Relative gene expression (expressed as fold difference of each treatment relative to the average of control treatment) was calculated using the 2^-ΔΔCt ^method, and the average calculated for each group. Separate calculations were made using 18 S and neuron-specific enolase as the reference 'housekeeping' gene.

### Statistical analysis

Statistical comparisons were analyzed using one-way analysis of variance ANOVA with *post-hoc *testing for comparison of multiple sets (Bonferonni's multiple comparison test). Paired t-tests were used for analysis of data where values represented paired observations. Values are expressed as the mean ± standard error of the mean (SEM) from at least three independent experiments, unless otherwise stated. Differences at *P *< 0.05 were considered as statistically significant. Statistical analyses were carried out with GraphPad Prism version 4.0 for Windows, GraphPad Software, San Diego, California, USA.

## Results

### Pioglitazone exposure protects cortical neurons from NO-mediated injury

**Figure 1:**
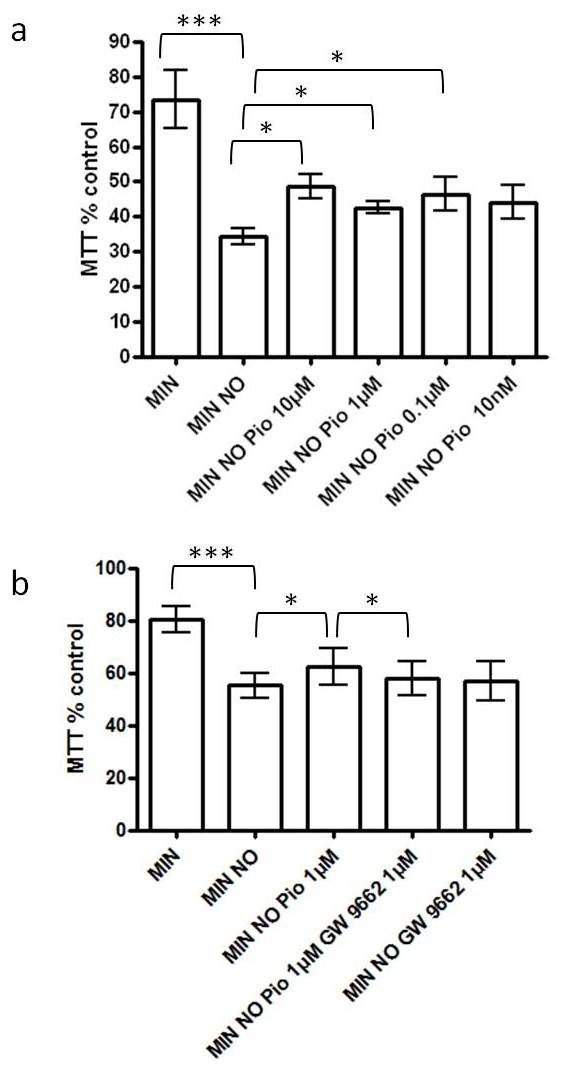
**Effect of pioglitazone on DETANONOate-mediated reductions in neuronal survival in cortical neuronal cultures**. (**a**) The effect of DETANONOate exposure (MIN NO) on cortical neuronal cell viability *in vitro *compared to serum free minimal media (MIN); and the effect of different concentrations (10 nM to 10 μM) of peroxisome proliferator activated receptor-γ (PPAR-γ) agonist, pioglitazone (Pio) on cortical neuronal cell viability *in vitro *(***P < 0.001 compared to MIN and *P < 0.05 compared to MIN NO; n = 5). Cultures were pre-treated with pioglitazone for 1 hour, prior to exposure to DETANONOate. (**b**) The effect of pre-treatment with the specific PPAR-γ antagonist, GW9662 (1 μM) on cortical neuronal viability *in vitro *compared to the effect of pioglitazone (1 μM) (***P < 0.001 compared to MIN, *P < 0.05 compared to MIN NO and *P < 0.05 compared to MIN NO Pio 1 μM). Cultures were treated with GW9662 for 1 hour prior to incubation with pioglitazone and DETANONOate. Cell viability was assessed by MTT assay. Data are expressed as percentage of cells grown in B27. Statistical significance was obtained by one-way ANOVA followed by a Bonferroni *post-hoc *test. ANOVA, analysis of variance.

To determine whether the PPAR-γ agonist pioglitazone could protect against the deleterious effects of a NO donor, cortical neuronal cultures were incubated for 1 hour in the presence of increasing concentrations (10 nM to 100 μM) of pioglitazone prior to exposure to DETANONOate (0.1 mM) for 24 hours. Cell viability was evaluated by assessing the reduction of the tetrazolium salt MTT, providing a general index of the metabolic state. Neuronal viability was significantly reduced when compared to control after nitric oxide exposure (Figure 1a). Preliminary dose response experiments determined the range of concentrations which appeared to have beneficial effects. In the presence of a range of concentrations (10 μM, 1 μM and 0.1 μM) of pioglitazone, neuronal viability was significantly increased during exposure to nitric oxide (Figure 1a). To examine whether the neuroprotection afforded by pioglitazone was a receptor-dependent effect, neurons were exposed to the specific PPAR-γ antagonist GW9662 (1 μM) for 1 hour, prior to incubation with pioglitazone (1 μM). As expected, neuronal viability was significantly increased following exposure to pioglitazone and this effect was abrogated in the presence of GW9662 (Figure 1b). Neuronal viability was not significantly different following exposure to the antagonist alone (Figure 1b). On the basis of these results, the concentrations of pioglitazone used to further study the effects of PPAR-γ activation on neuronal survival were determined. The final concentration of DMSO (vehicle) was always less than 0.1% and had no effects on cell viability (data not shown).

### Pioglitazone is neuroprotective and increases axonal length in the presence of LPS and cytokine-stimulated microglia

**Figure 2:**
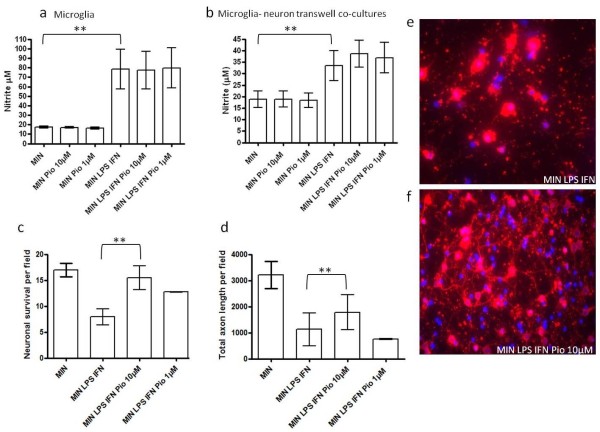
**Effect of pioglitazone on microglial-induced neuronal and axonal loss in transwell co-culture**. **(a) **Effect of pioglitazone (Pio; 1 and 10 μM) on nitrite production by microglial cultures either in the absence (MIN) or presence of LPS and IFN-γ (MIN LPS IFN). Cultures were exposed to IFN and LPS for 48 hours and nitrite levels measured in the culture supernatant (μM nitrite, **P < 0.01 compared to MIN; statistical significance was obtained by one-way ANOVA followed by Bonferroni post-hoc test. (**b**) Effect of pioglitazone (Pio; 1 and 10 μM) on nitrite production by neuron-microglia transwell co-cultures either in the absence (MIN) or presence of LPS and IFN-γ (MIN LPS IFN). Cultures were exposed to IFN and LPS for 48 hours and nitrite levels measured in the culture supernatant (μM nitrite, **P < 0.01 compared to MIN; statistical significance was obtained by one-way ANOVA followed by Bonferroni post-hoc test). (**c**) Effect of pre-treatment with pioglitazone (1 and 10 μM) on cortical neuronal survival in the presence of LPS and IFN-γ activated microglia in transwell co-culture (number of β-III tubulin cells per field) (**P < 0.01 compared to MIN LPS IFN, Student's *t*-test). (**d**) Effect of pre-treatment with pioglitazone (1 and 10 μM) on total axon length (**P < 0.01 compared to MIN LPS IFN, Student's *t*-test) in the presence of LPS and IFN-γ activated microglia in transwell co-culture (length of SMI-312 axons per field). Photomicrographs showing immunoreactivity for β-III tubulin (red) and DAPI (blue) in neuron-microglia transwell co-cultures in the presence of MIN LPS and IFN-γ (MIN LPS IFN) (**e**) and (**f**) MIN LPS IFN-γ and pioglitazone (10 μM) (MIN LPS IFN Pio 10 μM). ANOVA, analysis of variance; DAPI, 4',6-diamidino-2-phenylindole; IFN, interferon; LPS, Lipopolysaccharide. Microglial derived-nitric oxide has been shown to mediate significant neuronal and axonal loss *in vitro*. We examined the influence of pioglitazone pre-treatment on neuronal survival and axonal morphology in neuronal-microglial transwell co-cultures. Co-cultures were fixed and stained for βIII tubulin, SMI312 and the nuclear marker DAPI. Pre-treatment with pioglitazone (10 μM) conferred a neuroprotective effect with a significant increase in neuronal (Figure 2c) and total axon survival (Figure 2d) in transwell co-cultures of neurons and activated microglia. These data suggest that pioglitazone provides protection for cortical neurons in neuron-microglia co-cultures through mechanisms which may be independent of its effects on nitrite reduction and that cell-to-cell contact is not required for this to occur.

To determine the effect of pioglitazone on microglial NO generation, the levels of nitrite production were measured in microglia cultured alone or in transwell co-cultures with cortical neurons (allowing for the exchange of factors between microglia and neurons, but no direct cell-cell contact). Two different doses of pioglitazone (1 μM and 10 μM) were administered to neuronal-microglial transwell cultures and microglia cultured alone, 1 hour before LPS (1 μg/ml) and IFN (100 U/ml) treatment. LPS and IFN induced a significant increase in NO generation after 48 hours (Figure 2a and 2b). However, pre-treatment with pioglitazone failed to elicit a significant reduction in nitrite production by microglia cultured alone (Figure 2a) or in transwell co-cultures (Figure 2b).

### Induction of PPAR-γ and catalase activity by pioglitazone protects cortical neurons against inflammatory mediators

**Figure 3:**
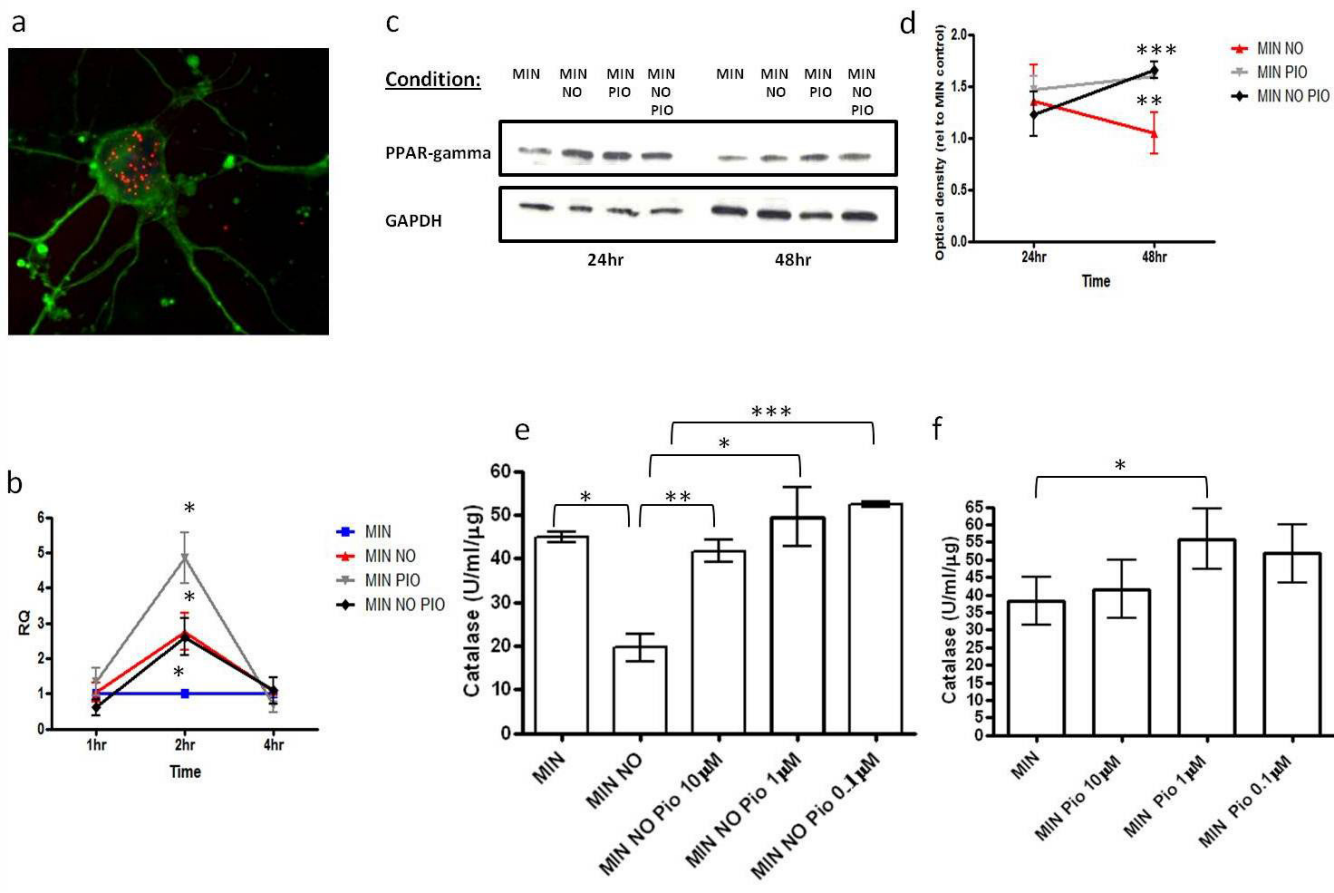
**Effect of pioglitazone on PPAR-γ expression and catalase activity in primary cortical neurons**. (**a**) Photomicrograph showing immunoreactivity for PPAR-γ (red), βIII tubulin (green) and DAPI (blue). PPAR-γ immunoreactivity shows a distinct punctuate nuclear staining pattern. PPAR-γ mRNA levels were measured by RT-PCR in (**b**) primary cortical neurons exposed to serum free minimal medium (MIN), minimal medium plus DETANONOate (0.1 mM; MIN NO), pioglitazone (1 μM) (MIN PIO) or DETANONOate (0.1 mM) plus pioglitazone (1 μM; MIN NO PIO). The fold difference relative to the average in control treated cells (MIN) was calculated for each treatment by the 2^-ΔΔCt ^method, with 18 S and neuron-specific enolase as endogenous controls. Plots depict the levels of transcript for PPAR-γ in cortical neurons following exposure to experimental conditions for 1, 2 and 4 hours (*P < 0.05 compared to MIN for MIN NO, MIN PIO and MIN NO PIO at 2 hours, Student's *t*-test). (**c**) Immunoblotting of PPAR-γ and loading control GAPDH in primary cortical neurons exposed to serum free minimal medium (MIN), minimal medium plus DETANONOate (0.1 mM; MIN NO), pioglitazone (1 μM) (MIN PIO) or DETANONOate (0.1 mM) plus pioglitazone (1 μM; MIN NO PIO) for 24 and 48 hours. (**d**) Western blot densitometric analysis of PPAR- γ expression in cortical neurons derived from experimental conditions outlined in (c) Data are given using arbitrary units of integrated density relative to MIN control (***P < 0.001, **P < 0.01 compared to MIN for MIN PIO and MIN NO PIO respectively at 48 hours, Student's *t*-test). (**e**) Effect of pioglitazone (Pio; 0.1 to 10 μM) on catalase enzymatic activity in cortical neurons exposed to DETANONOate (0.1 mM for 24 hours; MIN NO) (*P < 0.05 compared to MIN and *P < 0.05, **P < 0.01, ***P < 0.001 compared to MIN NO, Student's *t*-test). (**f**) Effect of pioglitazone (Pio; 0.1 to 10 μM) on catalase enzymatic activity in cortical neurons exposed to serum free minimal medium (MIN) (*P < 0.05, compared to MIN, Student's *t*-test). Data are expressed as mean ± SEM from at least three separate experiments. DAPI, 4',6-diamidino-2-phenylindole; PPAR-γ, peroxisomal proliferator activated receptor γ; SEM, standard error of the mean. Furthermore, we examined whether pioglitazone up-regulated the activity of catalase, a specific peroxisomal enzyme, using a commercial assay kit. Neurons treated with DETANONOate show a significant decrease in activity levels. However, pre-treatment with pioglitazone (10 μM, 1 μM and 0.1 μM) in the presence of DETANONOate (0.1 mM) elicited a significant increase in catalase activity compared to treatment with DETANONOate (0.1 mM) alone (Figure 3e). Furthermore, pioglitazone exposure alone (1 μM) elicited a significant increase in catalase levels (Figure 3f).

To further elucidate neuroprotective mechanisms of pioglitazone, we examined whether pioglitazone up-regulates neuronal expression of PPAR-γ at the transcript and protein level. Under normal conditions, PPAR-γ was localized to the nucleus of cortical neurons and exhibited a punctuate staining pattern (Figure 3a). Cortical neurons were incubated with pioglitazone (1 μM) in the presence or absence of DETANONOate (0.1 mM) for 1, 2 and 4 hours. Incubation with pioglitazone induced a significant increase in PPAR-γ mRNA levels at 2 hours, and also in neurons that were also exposed to DETANONOate. Interestingly, exposure to DETANONOate alone (0.1 mM) also elicited a significant increase in PPAR-γ levels with no apparent additive effect of DETANONOate and pioglitazone (Figure 3b). These changes were independent of the 18 S neuron specific enolase levels, used as endogenous controls. The effects of pioglitazone on PPAR-γ mRNA levels were paralleled by a significant rise in PPAR-γ protein levels over a 48-hour period, as evaluated by western blot analysis (Figure 3c, d).

### Enhancement of endogenous peroxisomal proliferation by pioglitazone protects against hydrogen peroxide-mediated neuronal injury

**Figure 4:**
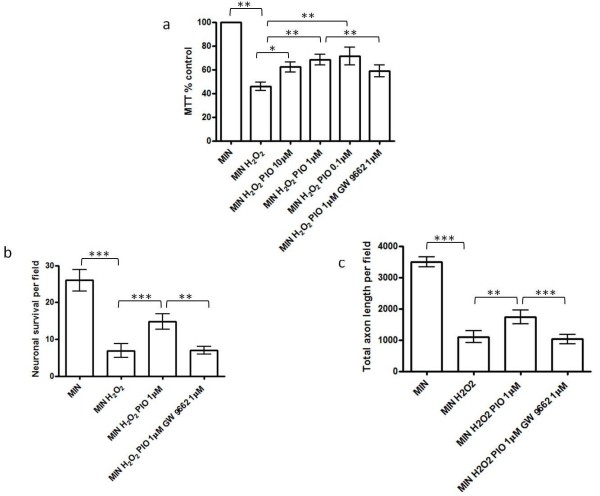
**Effect of pioglitazone on hydrogen peroxide-mediated reductions in neuronal survival in cortical neuronal culture**. (**a**) The effect of hydrogen peroxide exposure (MIN H_2_O_2_) (250 μM) on cortical neuronal viability *in vitro *compared to serum free minimal media (MIN) (**P < 0.01 compared with MIN); the effect of pioglitazone (0.1 μM to 10 μM; MIN H_2_O_2 _PIO) on cortical neuronal viability exposed to H_2_O_2 _(*P < 0.05 and **P < 0.01 as compared with MIN H_2_O_2_); and the effect of the PPAR-γ antagonist, GW9662 (1 μM) on pioglitazone-induced neuroprotection from H_2_O_2 _(**P < 0.01 comparing MIN H_2_O_2 _Pio 1 μM GW9662 1 μM with MIN H_2_O_2 _Pio 1 μM). Cultures were treated with GW9662 for 1 hour prior to incubation with pioglitazone for 1 hour followed by exposure to hydrogen peroxide. Cell viability was assessed by MTT assay. Data are expressed as percentage of cells grown in MIN medium. Statistical significance was obtained by one-way ANOVA followed by Bonferroni post-hoc test. (**b**) Effect of hydrogen peroxide exposure (250 μM; MIN H_2_O_2_) on cortical neuronal cell survival (number of βIII-tubulin cells per field; ***P < 0.001 as compared with MIN, Student's *t*-test); the effect of pioglitazone (1 μM) on cortical neuronal survival exposed to H_2_O_2 _(***P < 0.001 compared with MIN H_2_O_2_); and the effect of the PPAR-γ antagonist, GW9662 (1 μM) on pioglitazone-induced neuroprotection from H_2_O_2 _(**P < 0.01 comparing MIN H_2_O_2 _Pio 1 μM GW9662 1 μM with MIN H_2_O_2 _Pio 1 μM). (**c**) Effect of hydrogen peroxide exposure (250 μM H_2_O_2_) on axon length within neuronal cultures (determined by SMI312 staining; ***P < 0.001 as compared with MIN, Student's *t*-test); the effect of pioglitazone (1 μM) on axon length in neurons exposed to H_2_O_2 _(**P < 0.01 compared with MIN H_2_O_2_); and the effect of the PPAR-γ antagonist, GW9662 (1 μM) on pioglitazone-induced axon protection from H_2_O_2 _(***P < 0.001 comparing MIN H_2_O_2 _Pio 1 μM GW9662 1 μM with MIN H_2_O_2 _Pio 1 μM). Values represent the mean ± SEM from at least three separate experiments. ANOVA, analysis of variance; MTT, 3-(4,5-dimethyl-thiazol-2-yl)-2,5-diphenyltetrazolium bromide; PPAR-γ, peroxisomal proliferator activated receptor γ; SEM, standard error of the mean. To further examine the neuroprotective properties of pioglitazone, neurons were exposed to identical test conditions and cultures were then fixed and stained for βIII tubulin, SMI312 and the nuclear marker DAPI. The numbers of viable neurons (determined by nuclear appearance) expressing βIII tubulin were counted for each condition. As expected, neuronal survival was significantly increased following exposure to pioglitazone (1 μM) (Figure 4b). In addition, we examined the influence of pioglitazone on axonal morphology in these cultures. Neurofilament phosphorylation, as identified by the antibody SMI312, was examined as a marker of axonal integrity and length. Pre-treatment with pioglitazone (1 μM) caused a significant increase in total axon survival (Figure 4c). Pre-treatment with GW9662 led to a significant reduction in the neuroprotective (Figure 4b) and axonoprotective (Figure 4c) effects of pioglitazone. To determine further the role of catalase in pioglitazone-induced neuroprotection, we pre-incubated the cultures with the catalase inhibitor 3-amino triazole (10 mM) (3-AT) for 1 hour, prior to incubation with H_2_O_2 _(250 μM). The neuroprotective and axonoprotective effects of pioglitazone were attenuated by the addition of 3-AT (10 mM) (Figure 5a-5c). Furthermore, addition of exogenous catalase (100 U/ml) to cultures exposed to H_2_O_2 _improved neuronal survival (measured by MTT assay (Figure 5a) and neuronal morphology (Figure 5b) and axonal length within cultures (Figure 5c).

During inflammation, a major function of the peroxisomal enzyme catalase is to convert hydrogen peroxide (H_2_O_2_) into water. Therefore, having demonstrated induction of catalase activity by pioglitazone we wished to determine whether the pioglitazone could also protect cortical neurons against H_2_O_2_-mediated injury. Neuronal cultures were incubated for 1 hour in the presence of increasing concentrations of pioglitazone (0.1 to 10 μM) prior to exposure to hydrogen peroxide (250 μM). Neuronal viability was significantly reduced when compared to control after hydrogen peroxide exposure (250 μM for 24 hours; Figure 4a). In the presence of pioglitazone (10 μM, 1 μM and 0.1 μM), neuronal viability was significantly increased during exposure to hydrogen peroxide (Figure 4a). Furthermore, the neuroprotective effects of pioglitazone were prevented by the concomitant presence of GW9662 (1 μM) suggesting that PPAR-γ activation is necessary for the pioglitazone-induced neuroprotection of cortical neurons against the toxic effects of hydrogen peroxide.

**Figure 5:**
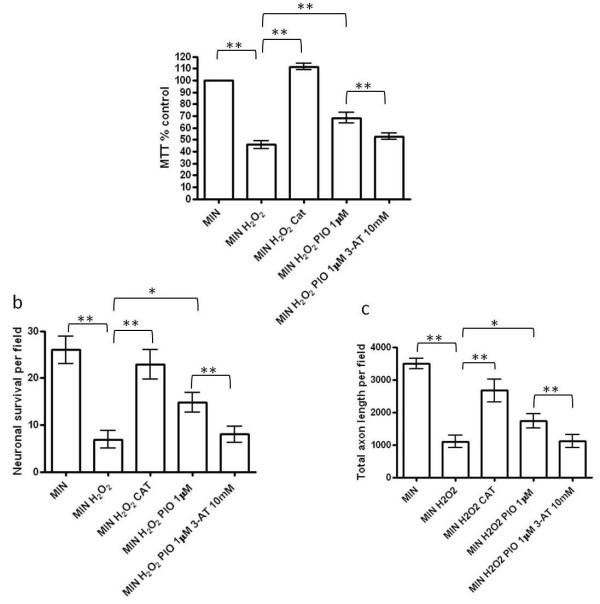
**Inhibition of catalase activity attenuates pioglitazone-induced protection from H_2_O_2 _in cortical neuronal cultures**. (**a**) The effect of hydrogen peroxide exposure (MIN H_2_O_2_; 250 μM) on cortical neuronal viability *in vitro *compared to serum free minimal media (MIN) (**P < 0.01 as compared with MIN); the effect of pioglitazone (1 μM) and the specific catalase inhibitor, 3-aminotriazole (3-AT) (10 mM) (**P < 0.01 MIN H_2_O_2 _compared to MIN H_2_O_2 _Pio 1 μM; and **P < 0.01 MIN H_2_O_2 _Pio 1 μM compared to MIN H_2_O_2 _Pio 1 μM 3-AT 10 mM) and the effect of catalase exposure (MIN H_2_O_2 _CAT; 100 U/ml) (**P < 0.01 MIN H_2_O_2 _compared to MIN H_2_O_2 _CAT) on cortical neuronal viability. Cultures were treated with pioglitazone for 1 hour prior to incubation with 3-AT for 1 hour followed by exposure to hydrogen peroxide. Cultures were treated with catalase for 1 hour prior to exposure to hydrogen peroxide. Cell viability was assessed by MTT assay. Data are expressed as percentage of cells grown in MIN medium. Statistical significance was obtained by one-way ANOVA followed by Bonferroni post-hoc test. (**b**) Effect of hydrogen peroxide exposure (250 μM H_2_O_2_) on cortical neuronal cell survival (number of βIII-tubulin cells per field; **P < 0.01 as compared with MIN, Student's *t*-test); the effect of pioglitazone (1 μM) on cortical neuronal viability exposed to H_2_O_2 _(*P < 0.05 compared with MIN H_2_O_2_); the effect of catalase exposure (MIN H_2_O_2 _CAT; 100 U/ml) (**P < 0.01 MIN H_2_O_2 _compared to MIN H_2_O_2 _CAT) and the effect of the specific catalase inhibitor, 3-AT (10 mM) on pioglitazone-induced neuroprotection from H_2_O_2 _(**P < 0.01 comparing MIN H_2_O_2 _Pio 1 μM with MIN H_2_O_2 _Pio 1 μM 3-AT 10 mM). (**c**) Effect of hydrogen peroxide exposure (250 μM H_2_O_2_) on axon length within neuronal cultures (determined by SMI312 staining; **P < 0.01 as compared with MIN, Student's *t*-test); the effect of pioglitazone (1 μM) on axon length in neurons exposed to H_2_O_2 _(*P < 0.05 compared with MIN H_2_O_2_); the effect of catalase exposure (MIN H_2_O_2 _CAT; 100 U/ml) (**P < 0.01 MIN H_2_O_2 _compared to MIN H_2_O_2 _CAT) and the effect of the specific catalase inhibitor, 3-AT, (10 mM) on pioglitazone-induced axon protection from H_2_O_2 _(**P < 0.01 comparing MIN H_2_O_2 _Pio 1 μM with MIN H_2_O_2 _Pio 1 μM 3-AT 10 mM). Values represent the mean ± SEM from at least three separate experiments. ANOVA, analysis of variance; MTT, 3-(4,5-dimethyl-thiazol-2-yl)-2,5-diphenyltetrazolium bromide; SEM, standard error of the mean.

## Discussion

In this study, we have performed a series of experiments demonstrating that the PPAR-γ agonist, pioglitazone is capable of promoting the survival of cortical neurons in the presence of inflammatory mediators *in vitro*, including microglia-induced injury in neuron-microglia transwell cultures. In addition, we have demonstrated that pioglitazone may have axonoprotective effects *in vitro*. Furthermore, this compound produced a two-fold increase in the specific activity of the peroxisomal enzyme catalase, together with a significant induction of PPAR-γ gene expression, in the presence of a NO donor, suggesting that modulation of PPAR-gamma activity and peroxisomal function by pioglitazone may contribute to improved neuronal survival.

Inflammation plays a pivotal role in the pathogenesis of several neurological disorders [39]. Activated microglia are capable of generating vast amounts of oxidizing radicals such as superoxide, hydrogen peroxide and nitric oxide [40] as well as proinflammatory mediators such as proteases and arachidonic acid derivatives which are all capable of eliciting tissue damage in the central nervous system [41]. It has been previously demonstrated that, at certain concentrations, the NO donor, DETANONOate is directly neurotoxic [5,6] and that microglial-derived NO significantly reduces the number of SMI312-positive axons per surviving neuron [6].

The role of PPAR-γ in regulating immune function has been extensively investigated, with a previous study demonstrating that pioglitazone can alleviate inflammation in experimental autoimmune encephalomyelitis (EAE) and reduce clinical severity [27]. Furthermore, previous reports have suggested that PPAR-γ agonists may reduce NO production by cultured microglial cells *in vitro *[42-44]. In the current study, however, pioglitazone did not have any inhibitory effect on NO production in IFN-γ/LPS activated microglia cultured alone or when neurons and microglia were separated with the use of transwell co-cultures. Despite the failure of pioglitazone to induce downregulation of NO production under these experimental conditions, pioglitazone (10 μM) significantly protected cortical neurons from injury by IFN-γ/LPS activated microglia in transwell co-culture. The neuroprotective effect of pioglitazone in transwell co-cultures cannot, therefore, be explained by the reduction in microglial activation commonly observed when PPAR-γ agonists are used [42-44], and suggests that the major effects of pioglitazone may occur through a primary protective effect specifically on neurons. However, the possibility should be considered that pioglitazone may exert an inhibitory effect on the microglial secretion of inflammatory mediators which may contribute to the observed neuroprotective effects [43].

Activated microglia are capable of causing widespread tissue damage in neuroinflammatory disorders [45,46] through their generation of vast amounts of oxidizing radicals, such as superoxide, hydrogen peroxide and nitric oxide [40]. In addition to demonstrating a protective effect of pioglitazone on neurons, we examined whether this drug could influence axonal morphology in the presence of activated microglia. Neurofilament phosphorylation is vital in the maintenance of axon stability and dephosphorylation of neurofilaments occurs within axons of inflammatory disorders such as multiple sclerosis leading to subsequent axon degeneration [47]. Our results demonstrate that microglial derived NO significantly reduces the total number of SMI-312-positive axons per field and that pioglitazone attenuates this severe microglia derived NO-mediated axon destruction.

Interestingly, when NO is secreted, it can intereact with O_2_^- ^to form peroxynitrite (ONOO^-^), an anion with strong oxidant properties [48]. Anti-oxidant enzymes such as Cu/Zn superoxide dismutase (SOD), Mn SOD and catalase (the latter being mainly localized to peroxisomes), work in concert to detoxify superoxide radicals into water and oxygen [49].We found that activation of PPAR-γ with neuroprotective doses of pioglitazone upregulated the expression of the PPAR-γ receptor and the activity of catalase, thus providing cortical neurons with a higher anti-oxidant capability. These results are consistent with the ability of PPAR-γ agonists to upregulate the levels of anti-oxidant enzymes in other experimental paradigms [49-52]. Furthermore, it is known that catalase contains functional PPAR-γ response elements in its promoter region [36], thus explaining the upregulation of this enzyme by pioglitazone.

In addition, our study confirmed a previous report [15] that cortical neurons express PPAR-γ, with its immunoreactivity being clearly localized to the nucleus. Neuroprotective doses of pioglitazone elicited a five-fold increase in the transcription of PPAR-γ, suggesting the existence of a positive feedback mechanism by which pioglitazone could sustain the responsiveness of cortical neurons by increasing the expression of its receptor. Interestingly, this early rise in PPAR-γ gene transcription was seen in neurons also exposed to the NO donor, suggesting that the effect of PPAR-γ agonism on PPAR-γ gene expression can occur independently of co-signalling with inflammatory mediators.

Anti-oxidant enzymes such as catalase and glutathione peroxidase work in concert to detoxify hydrogen peroxide into water and oxygen. We have demonstrated that neuroprotective doses of pioglitazone elicited a significant increase in activity of catalase. Therefore, in addition, we examined whether pioglitazone could also protect cortical neurons against the neurotoxic effects of hydrogen peroxide. As expected, pioglitazone (1 μM), protected cortical neurons from injury by hydrogen peroxide. Furthermore, our results demonstrate for the first time, that hydrogen peroxide reduces the total number of SMI312-positive axons, and that pioglitazone attenuates this severe H_2_O_2_-mediated axon destruction. Neuroprotection afforded by pioglitazone against both NO and H_2_O_2 _was abrogated by the presence of the specific PPAR-γ antagonist, GW9662. This is in accordance with a previous study whereby pioglitazone was found to exert its effects via a receptor-dependent mechanism [49].

## Conclusions

Taken together, our results show that activation of PPAR-γ by pioglitazone protects against neuronal cell death and axonal damage in *in vitro *models of inflammation, and provides further evidence of the neuroprotective effect provided by enhancement of endogenous peroxisomal activity. Further studies using *in vivo *models of neuroinflammation are justified to investigate the novel role of the PPARs in the prevention of the neuronal and axonal damage.

## Abbreviations

3-AT: 3-aminotriazole; ANOVA: Analysis of variance; BSA: Bovine serum albumin; CAT: Catalase; DAPI: 4',6-diamidino-2-phenylindole; DIV: days *in vitro*; D(MEM): Dulbecco's modified Eagle's medium; DMSO: Dimethyl sulfoxide; EAE: Experimental autoimmune encephalomyelitis; EDTA: Ethylenediaminetetraacetic acid; H_2_O_2_: Hydrogen peroxide; IFN: Interferon; LPS: Lipopolysaccharide; MTT: 3-(4,5-dimethyl-thiazol-2-yl)-2,5-diphenyltetrazolium bromide; NO: Nitric oxide; ONOO^-^: Peroxynitrite; PIO: Pioglitazone; PPAR: Peroxisome proliferator activated receptor; RNS: Reactive nitrogen species; ROS: Reactive oxygen species; SEM: Standard error of the mean; SOD: Superoxide dismutase; TZD: Thiazolidinediones; VLCFA: Very long chain fatty acids; X-ALD: X-linked adrenoleukodystrophy.

## Competing interests

The authors declare that they have no competing interests.

## Authors' contributions

EG, AW and NS conceived and designed the experiments. EG and MG performed the experiments. EG, KK, AW and NS analyzed the data. EG, AW and NS contributed reagents, materials and analysis tools.. EG wrote the paper. All authors read and approved the final manuscript.
